# Mechanisms of DNA Methyltransferase Recruitment in Mammals

**DOI:** 10.3390/genes9120617

**Published:** 2018-12-10

**Authors:** Marthe Laisné, Nikhil Gupta, Olivier Kirsh, Sriharsa Pradhan, Pierre-Antoine Defossez

**Affiliations:** 1 Epigenetics and Cell Fate, UMR7216 CNRS, University Paris Diderot, Sorbonne Paris Cité, 75013 Paris, France; marthe.laisne@gmail.com (M.L.); nikhilsspp@gmail.com (N.G.); olivier.kirsh@univ-paris-diderot.fr (O.K.); 2 New England Biolabs, 240 County Rd, Ipswich, MA 01938, USA; pradhan@neb.com

**Keywords:** epigenetics, DNA methylation, DNA methyltransferases

## Abstract

DNA methylation is an essential epigenetic mark in mammals. The proper distribution of this mark depends on accurate deposition and maintenance mechanisms, and underpins its functional role. This, in turn, depends on the precise recruitment and activation of de novo and maintenance DNA methyltransferases (DNMTs). In this review, we discuss mechanisms of recruitment of DNMTs by transcription factors and chromatin modifiers—and by RNA—and place these mechanisms in the context of biologically meaningful epigenetic events. We present hypotheses and speculations for future research, and underline the fundamental and practical benefits of better understanding the mechanisms that govern the recruitment of DNMTs.

## 1. DNA Methylation: An Essential and Dynamic Epigenetic Mark

The activity of the genome, especially gene expression, is regulated by epigenetic marks. This regulation has to combine two seemingly incompatible objectives: first, the epigenetic marks should be stable enough to contribute to a cell identity that is maintained through the lifetime of the cell, and that is passed on to the daughter cell [1]. Second, the marks have to be flexible enough to allow plasticity [2]. This plasticity can be very local, for instance at the scale of one promoter when a gene is induced by a stimulus. It can also be global, during the large reprogramming events that occur in the zygote after fertilization, or in primordial germ cells as they erase their parental identity to be able to produce gametes [3].

Understanding the stability and the dynamics of epigenetic marks is therefore important to discover the fundamentals of genome activity; and for detection and potential correction of the epigenetic drift that accompanies aging, as well as abnormal epigenetic reprogramming that is an underlying cause of many diseases such as cancers [4]. Finally, from a practical standpoint, understanding the stability and dynamics of chromatin marks is useful to reprogram the epigenome [5]. Again, this can be done on a single-gene scale, or on whole-genome scale to reprogram cells, which constitutes the starting point of regenerative medicine [6].

DNA methylation is a chromatin mark that is essential in mammals. It can rightly be called an “epigenetic” mark, as it has been proven to pass from mother to daughter cells, and sometimes even from one organismal generation to the next [7,8]. The main type of DNA methylation observed in mammals is the methylation of position five of cytosine within a CpG dinucleotide. Non-CpG methylation does occur, for instance in the brain [9,10], but its dynamics and roles are less understood and will not be discussed further here.

**Figure 1:**
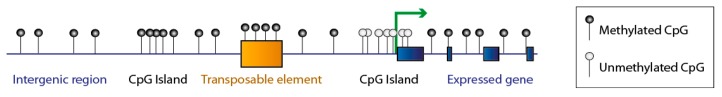
Genome-wide distribution of DNA methylation in mammalian species. DNA methylation occurs symmetrically on CpG sites: to simplify, only one DNA strand is shown on this figure. The mammalian genome has a low frequency of CpGs, but the majority of these are methylated (black lollipops) in intergenic regions, repeated elements and transposable elements, and in gene bodies. Conversely, CpG islands are rich in CpGs and are usually protected from DNA methylation (white lollipops). Unmethylated CpG islands frequently correspond to active promoters (green arrow).

In differentiated mammalian cells, about 80% of the CpGs in the genome are methylated; however, there are marked local differences (Figure 1). Intragenic regions and repeated elements are generally methylated. CpG islands, which are associated with the promoters of about two thirds of mammalian genes, are generally unmethylated; conversely methylated promoters are often silenced. Enhancers can also be dynamically methylated, which modifies their ability to recruit transcription factors and activate transcription [11,12,13]. Finally, the body of actively transcribed genes is methylated, and there is a positive correlation between expression and gene body methylation [14].

**Figure 2:**
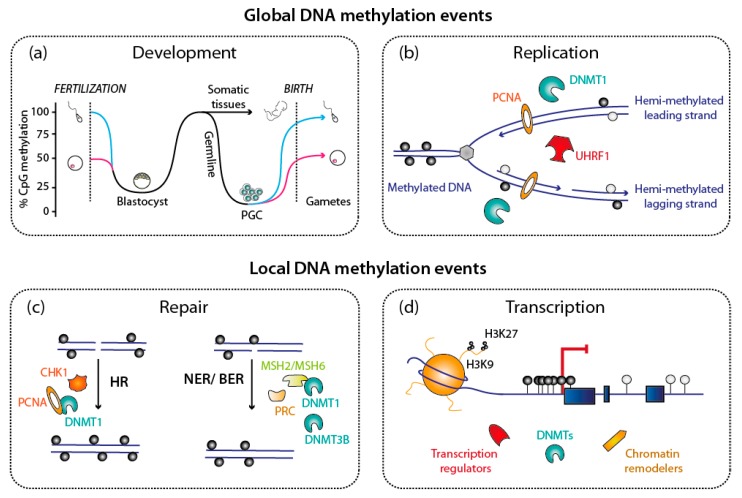
Biological processes involving DNA methylation, DNA methyltransferases (DNMTs) and keys partners. DNA methylation is involved in regulating many cellular processes; for all of them, DNMTs cooperate with several essential actors. (**a**,**b**) These DNA methylation events can affect the whole genome, as during the two methylation waves during mammalian life cycle, but also after DNA replicates at each cell cycle. (**c**,**d**) DNA methylation events can also be localized, as on the newly synthesized DNA after DNA repair. Selected examples, depicted in this cartoon, will be discussed further in this article. Abbreviations used: PGC: Progenitor germ cells; HR: Homologous Recombination; NER: Nucleotide Excision Repair; BER: Base Excision Repair.

DNA methylation is profoundly remodeled at several steps in the life of a mammalian organism (Figure 2). These steps include early development (Figure 2a), where the zygote after fertilization undergoes massive DNA demethylation, followed by widespread remethylation [15]. Another global demethylation wave occurs in primordial germ cells, as they erase parental imprints, before re-establishing the DNA methylation pattern found in gametes [3] (Figure 2a). The DNA methylation patterns are also globally challenged at each round of DNA replication (Figure 2b), as the newly synthesized DNA contains only unmethylated cytosines, which then have to be methylated if the parental pattern is to be maintained. Besides these genome-wide transitions, more local events are also observed: when DNA is damaged and repaired, the newly synthesized DNA is initially free of DNA methylation (Figure 2c). Also, local methylation on promoters occurs in the course of transcriptional regulation during development, or in response to a specific stimulus (Figure 2d).

The objective of this review is to summarize and discuss some of the mechanisms that are responsible for the stability and dynamics of DNA methylation, and therefore its functions. For the mark to occur, specific enzymes—the DNA methyltransferases (DNMTs) [16]—have to be recruited to target loci and become catalytically active. In this review, we will describe and discuss recent work on protein- and RNA-mediated recruitment of DNMTs, with a special emphasis on the mammalian enzymes, in the context of diverse functions DNA methylation plays in cellular processes and development.

## 2. Organization of the DNMTs and Its Functional Consequences

In this section, we will present an overview of the mammalian DNMTs: their domain organization, functions, and potential interacting regions with protein or RNA. The biological role of these interactions will be discussed in subsequent sections.

### 2.1. Several Non-Redundant Mammalian DNMTs Catalyze CpG Methylation

**Figure 3:**
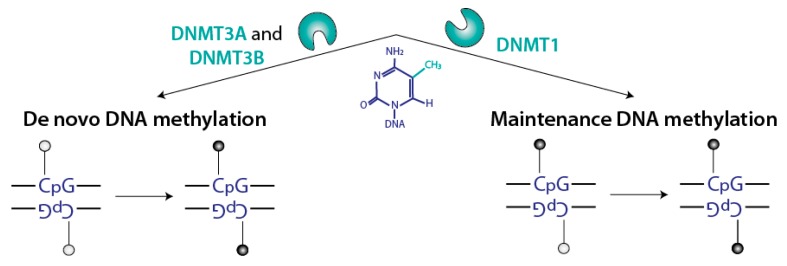
Specificities of the DNMTs DNA methylation can take place in two different contexts, and is processed by two distinct mechanisms: de novo DNA methylation involves fully unmethylated DNA and occurs on both strands, whereas maintenance of DNA methylation involves hemimethylated DNA.

**Figure 4:**
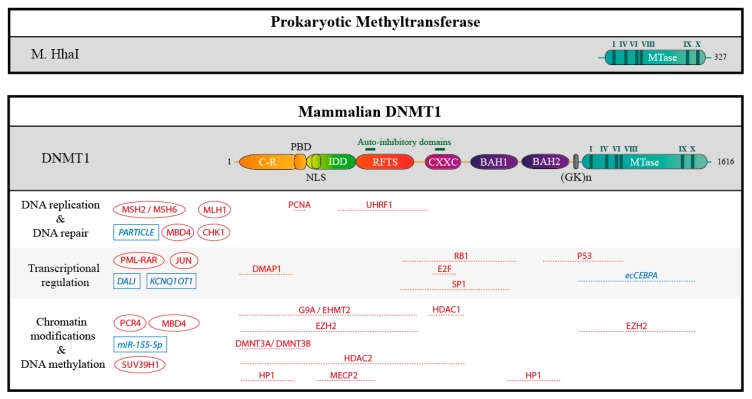
Schematic structure of the prokaryotic methyltransferase M. HhaI, compare to DNMT1 and partners. The human DNMT1 contains 1616 amino acids residues. The catalytic methyltransferase domain (MTase, in blue) is very similar to that of the prokaryotic methyltransferase M. HhaI and harbors highly conserved motifs (I-X, in dark blue). In addition, DNMT1 harbors a charge-rich (C-R) domain containing the proliferating cell nuclear antigen (PCNA) binding domain (PBD), an intrinsically disordered domain (IDD) with a nuclear localization sequence (NLS), a replication foci target sequence (RFTS), a zinc finger domain (CXXC), and two bromo-adjacent homology domains (BAH 1/2). The catalytic and the regulatory domains are connected by a series of Gly-Lys repeats. Auto-inhibitory domains are highlighted in green. In addition, some interacting proteins and RNAs are represented: if they are known, mapped interaction domains are indicated. Partners with unknown binding sites are shown on the left. Proteins are depicted in red, RNAs in blue.

**Figure 5:**
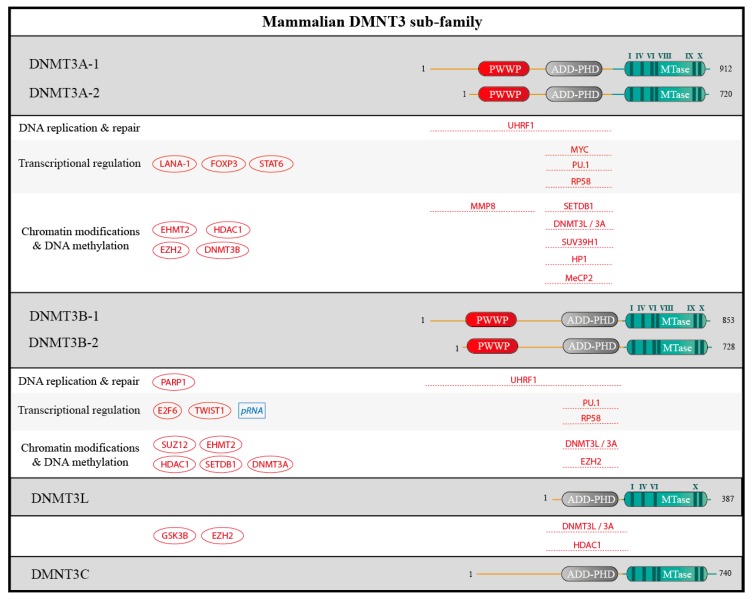
Schematic structure of DNMT3 sub-family and partners. Human DNMT3A, DNMT3B, DNMT3L and the rodent DNMT3C contain 912, 953, 387 and 740 amino acids residues, respectively and for the longest isoforms. For DNMT3A and DNMT3B, transcriptional isoforms due to alternative promoters are shown. The catalytic methyltransferase domain (MTase, in blue) harbors highly conserved motifs (I-X, in dark blue). DNMT3A and DNMT3B comprise a PWWP domain and an ATRX-DNMTB-DNMT3L and Plant-Homeodomain (ADD-PHD), that is also found in DNMT3L and DNMT3C. Interacting proteins and RNA are depicted as in Figure 4.

Cytosine methylation results from the covalent transfer of a methyl group from *S*-adenosyl methionine (SAM) to the carbon C-5 of cytosines to produce 5-methylcytosine (Figure 3). This activity is present in bacterial proteins, such as M. HhaI, as part of their restriction/modification systems [17], and iterative searches for mammalian proteins containing a domain similar to the bacterial enzymes led to the identification of the different DNMTs: DNMT1, DNMT2, DNMT3A, DNMT3B, DNMT3C, and DNMT3L (Figure 4 and Figure 5). DNMT3C is present only in rodents, whereas all mammals express the other proteins.

In spite of its similarity to DNA-modifying enzymes, DNMT2 proved to be a tRNA methyltransferase [18,19] and will not be discussed further. DMNT3C is specific to muroids, and was discovered very recently [20]. Our review will for the most part focus on the better-known, catalytically active, enzymes DNMT1, DNMT3A and DNMT3B. We will also discuss DNMT3L: this protein, even though it has no intrinsic catalytic activity, is necessary to stimulate the action of DNMT3A and DNMT3B [21].

Genetic studies in the mouse [22] and other organisms showed that the DNMTs are non-redundant. One reason for their uniqueness is a specific expression pattern [22], but other factors are also involved, as the enzymes have clearly different activities in vitro [23]. Remarkably, papers by Riggs [24] as well as Holliday and Pugh [25] proposed as early as 1975 that “maintenance” DNA methylation might be distinguished from “de novo” DNA methylation, and carried out by different proteins. These predictions were validated by experimental work in the subsequent decades: broadly speaking, the maintenance of DNA methylation on hemimethylated CpG sites (generated by DNA replication), is mostly due to DNMT1, which is expressed in all cycling cells; in contrast de novo DNA methylation on both strands of previously unmethylated CpG is mostly carried out by DNMT3A and DNMT3B (Figure 2). There are exceptions to this general division of labor [26,27], but this working model is useful.

### 2.2. DNMTs Have Divergent Non-Catalytic Domains

As said above, all vertebrate DNMTs share a conserved region necessary for catalysis; it permits the binding to SAM, and the recognition of DNA. This domain resembles that found in bacterial DNMTs such as M. HhaI, emphasizing their shared evolutionary origin (Figure 4 and Figure 5).

Besides this conserved catalytic domain, which is always found at the C-terminus, the different DNMTs contain divergent N-termini that differ in size, as well as in the number and nature of domains they contain. These N-terminal regions contribute to the non-redundant functions of DNMTs.

DNMT1 is the largest of the enzymes (1616 amino acids in humans). Its catalytic domain is separated from the large N-terminal regulatory region by a series of Lys-Gly dipeptide repeats. The N-terminal part harbors different domains: (i) a charge-rich (C-R) domain, which includes a proliferating cell nuclear antigen (PCNA) binding domain (PBD); (ii) an “intrinsically disordered domain”, found only in eutherian mammals [28,29], which contains a nuclear localization sequence (NLS); (iii) a replication foci target sequence (RFTS) domain; (iv) a zinc finger DNA binding domain (CXXC); (v) two bromo-adjacent homology domains (BAH1/2) [30].

Enzymes in the DNMT3 family have closely related architectures: DNMT3A and DNMT3B are composed of (i) the N-terminal domain, which is essential for DNA-binding; (ii) a PWWP domain which recognizes H3K36me3; (iii) an ADD-PHD domain (ATRX-DNMT3B-DNMT3L/plant homeodomain), which binds unmethylated H3K4; and (iv) the catalytic domain (Figure 5). Several isoforms of DNMT3A and DNMT3B, due to alternative promoters or splicing events, have been identified both in human and mouse, and could be involved in different functions. For example, the two major isoforms of DNMT3A shown in Figure 5 have different genomic localizations despite their high similarity [31]. The rodent-specific protein DMNT3C is similar to DMNT3B but lacks the PWWP domain [20]. DNMT3L resembles DNMT3C.

### 2.3. DNMTs Form Complexes

Many of the motifs described above mediate protein-protein interactions, allowing the various DNMTs to participate in multiprotein complexes.

DNMT3A can form hetero-oligomeric complexes: as a heteroduplex with DNMT3L, which increases the processivity of the enzyme, or as a linear heterotetramer with two DNMT3L subunits (at the edges of the tetramer) and two DNMT3A subunits [32]. Moreover, DNMT3A alone or DNMT3A/DNMT3L complexes can also cooperatively bind DNA, and form large multimeric DNA/protein fibers [33].

Besides homo- or hetero-oligomerization, biochemical approaches such as immunoprecipitation followed by mass spectrometry have revealed that the DNMTs, as other chromatin-modifying enzymes, are part of larger protein complexes [34,35,36]. Estimating the stoichiometry of these complexes can help reveal which members of the complex are constitutively associated to the DNMTs, and which are minor or transient interactors; this quantitative approach has been historically challenging, but emerging technologies should help improve the situation [37].

### 2.4. DNMTs Bind Nucleic Acids

The DNMTs contain several domains that can bind nucleic acids, i.e., DNA or RNA. The CXXC domain of DNMT1 is a type of zinc-finger with preferred binding to unmethylated (rather than methylated) CpGs, with an important role in the auto-inhibition mechanism [30]. The catalytic domain also contains a number of basic residues which interact in a sequence-independent manner with the negatively charged DNA backbone [38]. Interestingly, the DNMTs have an affinity for G-quadruplexes [39], and this is biologically relevant [40]. Finally, the PWWP of the DNMT3s has been shown to bind DNA [41].

### 2.5. DNMTs Are Autoinhibited

Finally, an important functional characteristic of the DNMTs is that they are intramolecularly inhibited, which presumably decreases their off-target activity. Structural and biochemical experiments have that DNMT1 is inhibited by intramolecular interactions between the catalytic site and the RFTD domain or the CXXC domain [5]. DNMT3A is also autoinhibited, albeit by a different domain, the ADD [42,43]. 

### 2.6. Functional Consequences for Recruitment Mechanisms

Four important conclusions can be drawn from this overview of the DNMTs. First, the enzymes have a large number of domains, structured or unstructured, with which to establish protein-protein or protein-nucleic acid interactions. Second, some of these domains engage in intramolecular interactions with the catalytic domain and inhibit its activity. Therefore, the recruitment of DNMTs by a protein or RNA interactor may have two separate effects: increasing the local enzyme concentration, but also activating the enzyme at its site of recruitment [42]. Third, the recruitment of a DNMT will not necessarily lead to local methylation: the interaction could in fact break up a catalytically productive complex, or stabilize the auto-inhibited form of the enzyme [44]. Fourth, while DNA methylation is of course the best-known activity of DNMTs, they might possess other important functions that are unrelated to DNA methylation. These functions could be intrinsic, or borne by DNMT interactors: it is clear, for instance, that DNMTs associate with other chromatin-modifying factors, such as histone deacetylases (HDACs) [45]. Therefore, recruiting a DNMT may alter DNA methylation locally, but it could also have other consequences on chromatin.

## 3. DNMT Recruitment in the Regulation of Chromatin Structure and Gene Expression

DNA methylation is deeply linked to cell identity, as it is a determinant of the cellular transcriptional program [46]. Besides regulating cellular gene expression, DNA methylation is also a key contributor to the transcriptional repression of transposons [21]. These functions of DNA methylation depend on the recruitment of DNMTs with transcription factors and other DNA binding proteins, with histone marks and chromatin modifiers, and with non-coding RNAs. We review these interactions in the subsequent sections, with an emphasis on the most recent data.

### 3.1. Interaction with Promoter-Bound Transcription Factors

It is well described that DNA methylation status can influence the recruitment of transcriptional regulators [47]. Conversely, transcription factors bound to DNA can also directly recruit the DNA methylation machinery. This was first reported for an oncogenic transcription factor, PML-RAR [48], but the paradigm was rapidly extended to unaltered cellular transcription factors, such as p53 recruiting DNMT1 to silence the *SURVIVIN* promoter [49], and MYC recruiting DNMT3a to silence *p21/CDKN1A* [50]. Since then, many other examples of cellular [46] or viral [47] transcription factors recruiting DNMTs to promoters, via direct interactions, have been discovered. This topic, however, has been discussed in a previous review [48]. Most reported examples concern the recruitment of DNMTs to promoter regions. Interestingly, a previous paper showed that the zinc finger protein ZBTB24, which is found mutated in Immunodeficiency, Centromere instability and Facial anomalies (ICF) syndrome, is likely to recruit DNMT3B to certain gene bodies [49]. This mechanism seems to apply not only to the genes transcribed by RNA Polymerase II (PolII), but also the genes that are targets of PolI [50] or PolIII [51] Also, while the recruitment of DNMTs seems to generally be accompanied by transcriptional repression, the mechanisms may be varied, at least for DNMT1. In some cases, methylation of DNA by the enzymes seems to be the cause of repression, while in others the enzyme may repress transcription independently of its catalytic activity [51]. This non-catalytic repression seems itself due to protein-protein interactions by which DNMT1 recruits chromatin-modifying enzymes [52].

Besides the relative contributions of catalytic and non-catalytic functions of DNMTs to promoter activity, several general questions await clarification. In a given cell, what fraction of the DNMT molecules is engaged in transcriptional regulation? What are the dynamics of these interactions, and are they regulated by modifications of the transcription factors, the DNMTs, or both? In all but a few examples [53], this regulation is unknown. Do the same mechanisms occur at enhancers [54]? Finally, how can the DNMTs interact with such a large number of unrelated transcriptional regulators? The situation is somewhat reminiscent of the transcription machinery, which can be recruited by many different, apparently unstructured transcriptional activation domains [55]. It would be of interest to determine whether the DNMTs also contain low-complexity regions that function in a similar manner.

### 3.2. Interaction with Chromatin Modifiers

DNMT1 has different histone-binding partners (histone-methyltransferases, histone deacetylases, but also nucleosome remodelers like SNF2H), mainly recruited through its N-terminal domain, and depicted in Figure 4. An illustrative example is the interaction between DNMT1 and the H3K9 methyltransferase G9a/EHMT2 [56], which helps coordinate DNA and histone methylation after DNA replication. More generally, this crosstalk between the DNA and H3K9 methylation pathways seems fairly prevalent [57], and may be of particular importance at repeated sequences such as centromeres [58].

Less is known about the interactome of DNMT3A and DNMT3B, but some of their chromatin-modifier partners have been identified: for example, DNMT3A interacts with the histone-lysine methyltransferase SETDB1 through its plant homeodomain (PHD) zinc finger and contributes to gene silencing [59]. The relationship between the Polycomb machinery and the DNA methylation machinery is probably more complex than initially thought [57]: while it has been ascertained that EZH2 can in fact recruit DNMT3A to the genome, this recruitment is not sufficient to trigger de novo DNA methylation [60]. The histone-binding protein MPP8 forms a molecular bridge between EHMT1/GLP and DNMT3A, which may help coordinate DNA methylation and H3K9 methylation [61]. The interaction involves the chromodomain of MPP8, which binds a methylated lysine in the N-terminus of DNMT3A [61]. A last example is that the ATRX domain of DNMT3A and the histone acetyltransferase HDAC1 can interact; consequently DNMT3A promotes histone deacetylation near the binding sites of its interactor RP58 [62].

### 3.3. Integrating Interaction Mechanisms to Dynamically Regulate a Transcriptional Program: The Example of Germline Genes

**Figure 6:**
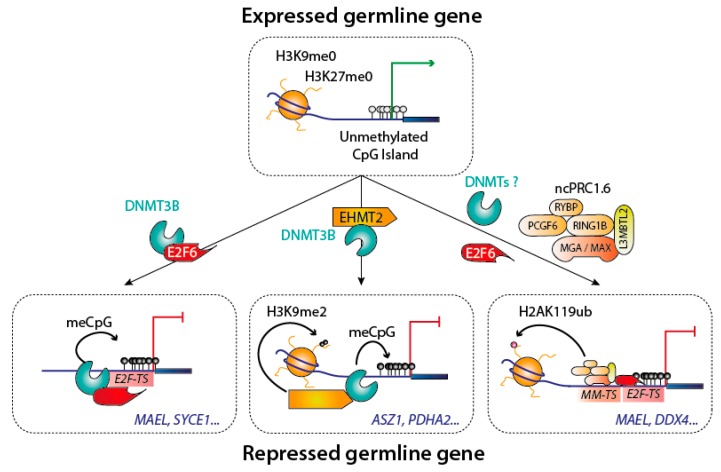
Silencing of the germline genes during implantation. Different complexes are involved in the silencing of germline genes. Three well documented mechanisms are shown, with some example of known targeted germline genes in blue. Abbreviations used: *E2F-TS*: E2F target sequence; *MM-TS*: MGA-MAX target sequence.

Genes specifically expressed in the germline permit the formation of gametes; examples of such genes are those necessary for meiosis. Their expression is tightly repressed in somatic cells, and it was recognized early on that many of these genes require DNA methylation for repression [63]. Genetic and molecular experiments showed that the methylation of germline genes is laid by DNMT3B [63], and that this deposition was crucially dependent on the transcription factor E2F6, which recruits DNMT3B to its target sites [64]. This transcription factor/DNMT interaction is one part of a complex web of regulation, as the histone modifying enzyme G9a/EHMT2 is also necessary for DNA methylation to occur on certain germline genes [65]. In addition, E2F6 also takes part in a parallel transcriptional repression mechanism, involving a non-canonical polycomb repressive complex 1 (PRC1) complex [66]. The germline genes therefore provide a clear example of the superposition of DNMT recruitment mechanisms and of repressive pathways [67] (Figure 6).

### 3.4. The Role of lncRNAs and miRNAs

Over the past decade, long non-coding RNAs (lncRNAs) have emerged as major regulators of the genome, and they act in part by recruiting nucleoprotein complexes [68]. Mouse and human DNMTs are among the chromatin factors that can be recruited by lncRNAs.

One illustrative instance of this principle was described at the mouse ribosomal DNA (rDNA) locus. There, promoter-associated RNAs (pRNAs), ~200 nucleotides in length, are formed and remain associated with the rDNA promoter via the formation of an RNA:DNA triplex [69]. Fascinatingly, they also associate with DNMT3B, promote its recruitment to the locus, and the inhibition of rDNA transcription [69]. To the best of our knowledge, the region of DNMT3B that is recruited by the pRNA has not been described. However, the recruitment by triplex-forming RNAs has now been shown for DNMT1 as well, as in the case of *PARTICLE*, a lncRNA induced by genotoxic insult [70,71].

Other cases of DNMT cis-recruitment by lncRNA do not seem to involve a triplex-formed RNA. For instance, the *KCNQ1* is a paradigmatic imprinted region, i.e., a region in which the alleles on the paternal and maternal chromosomes have a stable and reciprocal expression pattern. Imprinted genes often produce lncRNA, which participate in the allele-specific regulation of the locus [72]. It was observed that the antisense RNA *KCNQ1OT1*, produced by the paternal allele, interacts with DNMT1, and recruits it to the paternal chromosome, where expression is silenced [73]. The lncRNA-mediated recruitment of DNMTs may also function in trans, as shown with a regulator of neuronal development, *DALI* [74], or within the tumor-suppressive PTEN locus [75].

Although recruitment of DNMTs by RNA is thought to repress the target loci, a recent report challenges this notion [75]. In this paper, it is shown that thousands of loci produce lncRNA which associate with DNMT1. One specific lncRNA, starting upstream of *CEBPA* gene and named *ecCEBPA*, is studied in more detail, and found to adopt a stem-loop structure which binds with high affinity to the catalytic domain of DNMT1. Importantly, this lncRNA protects the *CEBPA* gene against methylation, again illustrating the principle that recruitment of DNMTs does not necessarily equate increased local DNA methylation.

It is noteworthy that microRNAs generated by the ribonuclease DROSHA have been recently shown to bind DNMT1 and decrease its activity [76]. At least one of them, *miR-155-5p*, can apparently act globally and decrease genome methylation [77]. Future work will hopefully reveal whether these mechanisms can also act in recruitment pathways.

### 3.5. Repression of Transposable Elements: The Role of piRNAs

During its evolution, the mammalian genome has been populated by many transposable elements (TEs). The majority of these sequences are now inactive due to accumulated mutations; however, a small number of copies still retain the potential to transpose. It is now accepted that these mobile elements have a positive role as “evolutionary drivers” [78,79,80]. Nevertheless, transposition is potentially harmful, and cells have evolved complex mechanisms to tightly control transposons, in part at the transcriptional level [81].

The transcriptional repression of transposons depends heavily on DNA methylation [21], and uses several protein-mediated DNMT recruitment mechanisms. For instance, repression of transposons by TRIM28 leads to DNA methylation [82], although the mechanistic details are lacking. But an original RNA-dependent recruitment pathway is also involved. Indeed, one of the most crucial mechanisms to silence the TEs during male gametogenesis is the PIWI/piRNA pathway in which the piRNAs (small RNAs of 25–32 nt) combine with the PIWI proteins from the Argonaute family to form piRISC complexes. These are directed in a RNA-directed manner to initiate the repression of TEs by recruiting histone modifiers and DNMTs.

PIWI proteins have a defined expression window, among them, MIWI2 expression coincides with the de-novo methylation wave in PGC and also has a nuclear localization, while another critical PIWI protein MILI has a broader expression window and is exclusively cytoplasmic. MIWI2 is proposed to recruit DNMTs, however a detailed mechanism for the recruitment of DNMTs is yet to be elucidated. Further, loss of function studies for MILI and MIWI2 indicate that they may have some non-overlapping roles in DNA methylation of TEs, thus highlighting unknown mechanisms for the recruitment of DNMTs at TEs [81,83,84]. Further, other studies postulate the role of piRNA mediated DNA methylation well beyond the TEs, in the regulation of mRNA transcripts in both somatic and germ cells, imprinted DMR locus and oncogenes involved in cancer [85,86,87].

## 4. Maintenance of DNA Methylation during DNA Replication

A very well described role of DNMT1 is to carry out maintenance DNA methylation following DNA replication. This is mediated by a number of well-characterized interactions, which are described in the following paragraphs.

### 4.1. Interaction with the DNA Replication Machinery

As noted above, it was realized early on that DNA replication would reset the genome to a hemimethylated state, and that if the marks were to be maintained stably through cell divisions, a maintenance mechanism must exist. It was therefore a momentous advance when DNMT1 was shown to directly contact PCNA, providing a direct molecular link between DNA replication and DNA methylation [88]. The exact interacting region was found by pull-down assays against different DNMT1 fragments [89]. It occurs through a conserved PCNA-interacting protein (PIP) motif, a motif which is found in many of the proteins that interact with PCNA and is usually located in intrinsically disordered regions of the proteins [90]. Interestingly, the inactivation of this motif clearly prevents the interaction of DNMT1 with PCNA, and impedes the recruitment of DNMT1 to replication foci in early and mid S phase, but it does not affect the steady-state level of DNA methylation in mouse ES cells [91]. In other words, the direct interaction with PCNA seems to facilitate the recruitment of DNMT1 to replication sites, but other mechanisms can compensate if this interaction is not permitted. We will touch on some of these mechanisms in the following sections.

### 4.2. Role of UHRF1 and Recognition of Modified Histones

Besides DNMT1, another protein is known to be critically required for DNA methylation maintenance: UHRF1 [92,93]. Initial models suggested that UHRF1 promotes DNA methylation maintenance by directly recruiting DNMT1 through the RFTS domain [94] and then activating the enzyme [95,96], but more recent work suggest that less direct mechanisms are also involved. In particular, UHRF1 is a ubiquitin ligase that can modify histones, and ubiquitinated histones can be bound by the RFTS domain of DNMT1 [97,98]. This important topic is covered in more detail elsewhere in this issue.

### 4.3. Unresolved Questions

There is no doubt that DNA methylation maintenance is, somehow, coupled to DNA replication. However, as new discoveries are made, it emerges that the simple and elegant model first reported—a direct interaction of DNMT1 with PCNA—coexists with others. Several important questions are still unanswered. The kinetics with which DNA methylation is re-established is a matter of controversy [99,100]. Are lncRNAs involved in DNA methylation maintenance? How much do the “de novo” methyltransferases contribute to maintenance activity, and how [26]? Are the mechanisms of DNA methylation maintenance identical on leading and lagging strand [101]? The answer to these questions is directly linked to the identification of mechanisms recruiting the respective enzymes to their targets.

## 5. Restoration of DNA Methylation after DNA Damage

After DNA damage, different types of DNA repair can take place: Nucleotide Excision Repair (NER), Base Excision Repair (BER), Non-homologous End Joining (NHEJ) and Homologous Recombination (HR). The choice depends on the type of damage (for instance, presence of adducts versus presence of a double-strand break), and on the position within the cell cycle, which determines whether a sister chromatid is present to serve as a template for repair. Some types of repair, such as HR, entail the resection and resynthesis of large sections of DNA (up to several kilobases in mammals [102]). Therefore, it is expected that the DNA methylation machinery should be recruited to sites of HR to permit the re-establishment of the mark on the newly re-synthesized DNA, and this prediction has indeed been verified experimentally for DNMT1, both by microscopy [103,104] and by biochemical assays [105,106]. It is likely that one mechanism of recruitment is the direct interaction with PCNA, as the kinetics of recruitment of PCNA and DNMT1 are similar, and a region containing the interaction motif is necessary for the recruitment to occur [103]. Nevertheless, it is possible that other recruitment mechanisms also take place; in fact direct interaction with the DNA damage response protein CHK1 has been shown, but has not been worked out in mechanistic detail [107]. Yet other mechanisms may exist: for instance, UHRF1 has been proposed to directly recognize certain types of damaged DNA [108,109], and it would be of interest to determine whether this permits the recruitment of DNMT1 in parallel to the PCNA-driven pathway.

A particular situation leading to DNA damage is oxidative stress [110]. Oxidized bases such as 8-oxo-G are repaired in large part by BER and NER, but not HR [111]. An interesting report showed that oxidative stress led to the formation of large complexes containing DNMT1, DNMT3B, and Polycomb proteins, which were then addressed to previously unmethylated CpG islands [112]. Follow-up work recently clarified the mechanism, which depends on the mismatch repair proteins MSH2/MSH6 [113,114], but not on PCNA. While the interaction of DNMT1 with MSH2/MSH6 is clearly stimulated by oxidative stress, its mechanistic underpinning remains to be precised: what are the domains of the proteins involved? Why is the interaction stimulated by oxidation? These results echo earlier findings showing an interaction of DNMT1 with the mismatch repair protein MLH1 [104].

To summarize, DNMTs are recruited to chromatin after several types of DNA damage, double-strand breaks and oxidative damage have both been proved directly, and it would be interesting to assess whether other lesions, such as single-strand breaks or pyrimidine dimers also have the same effect. One mechanism that is unambiguously involved is the interaction of DNMT1 with PCNA. Interactions with mismatch repair proteins also appear important, but their molecular basis is unclear. Needless to say, the future may reveal yet other modes of DNMT recruitment after DNA damage.

## 6. Conclusions and Perspectives

### 6.1. Conceptual Advances in the Roles of DNMTs

The development of high-throughput sequencing technologies has revolutionized our ability to map DNA methylation, to identify genomic loci bound by DNMTs, and to identify RNAs associated with DNMTs. In parallel, advances in mass spectrometry have also made it much easier to detect and quantify protein complexes, while new methods in microscopy give us insight into their location and dynamics. The combination of these methods has allowed the community to arrive at the cumulative knowledge presented in this review. Much of the evidence has cemented early insight that DNA methylation is a critical epigenetic mechanism, which contributes to cell identity by regulating transcriptional programs, by ensuring the proper chromatin composition on key chromosome elements such as centromeres, and by repressing repeated elements. Another important lesson is that DNA methylation is part of a complex mesh of chromatin regulation mechanisms, which includes non-coding RNAs, histone- and nucleosome-modifiers, and DNA demethylation activities [15]. An important issue, not discussed here, is how DNA methylation is coordinated to DNA demethylation to achieve the final patterns seen in cells [115].

### 6.2. Targeting the DNMTs for Epigenome Editing

Artificially recruiting DNMTs to a locus of interest has long been considered as potentially useful to “edit the epigenome”, for example to turn off the expression of oncogenes in tumor cells, or to remodel the genome of stem cells [116]. The idea has been made much easier to implement with the development of Cas9-based platforms [5,117]. The results described in this review have practical applications. First, they show that non-catalytic activities of DNMTs can be critical for repression and may have to be maintained for a Cas9 fusion to work efficiently. Second, they underline that increasing the local concentration of DNMTs by recruitment does not always translate into increased DNA methylation, as self-inhibitory mechanisms have to be overcome. Third, they show that RNA can be explored as a way to recruit DNMTs. Again, the recruitment can either lead to local DNA methylation and repression [85] or, conversely, to inhibition of the enzyme and local protection from its activity [118].

### 6.3. Consequences for Disease and Treatment

Most of the mechanisms we have described occur during the development and life of a healthy organism. We have already mentioned, however, that some of these mechanisms can be subverted by viral or oncogenic proteins. Understanding these events molecularly may help target them pharmaceutically to fight infections and cancers. More generally, the DNA methylation patterns drift during human aging, and this may contribute to the increase in cancer risk with age [119]. It will be of great interest in the future to determine if the DNMT recruitment mechanisms that have been identified go awry in aging cells, and whether this can be prevented or reversed pharmaceutically.
